# The prognostic value of *TP53* mutations in hypopharyngeal squamous cell carcinoma

**DOI:** 10.1186/s12885-017-3913-1

**Published:** 2017-12-28

**Authors:** Go Omura, Mizuo Ando, Yasuhiro Ebihara, Yuki Saito, Kenya Kobayashi, Osamu Fukuoka, Ken Akashi, Masafumi Yoshida, Takahiro Asakage, Tatsuya Yamasoba

**Affiliations:** 1 Department of Otolaryngology-Head and Neck Surgery, Faculty of Medicine, The University of Tokyo, 7-3-1 Hongo, Bunkyo-ku, Tokyo, 113-8655 Japan; 2 Department of Head and Neck Surgery, National Cancer Center Hospital, Tokyo, Japan; 3 Department of Head and Neck Surgery, Saitama Medical University International Medical Center, Saitama, Japan; 4 Department of Head and Neck Surgery, Faculty of Medicine, Tokyo Medical and Dental University, Tokyo, Japan

**Keywords:** *TP53* mutation, Hypopharyngeal squamous cell carcinoma, Truncating mutation, Prognosis, Pharyngectomy

## Abstract

**Background:**

*TP53* is the most frequently mutated gene in human cancers. Previous studies reported that *TP53* mutations correlated with poor prognoses in patients with head and neck squamous cell carcinoma (HNSCC). However, the relationship between *TP53* mutations and hypopharyngeal squamous cell carcinoma (HPSCC) is not known. The current study aimed to evaluate *TP53* mutation status as a predictive biomarker in patients with HPSCC.

**Methods:**

We retrospectively reviewed the clinical charts of 57 HPSCC patients treated with initial surgery between 2008 and 2014. *TP53* mutation status was determined by Sanger sequencing, and patients were classified into wild-type, missense mutation, and truncating mutation groups. Additionally, p53 expression was determined using immunohistochemistry in surgical specimens.

**Results:**

*TP53* mutations were identified in 39 (68%) patients. The 3-year disease-specific survival (DSS) rate of wild-type*,* missense mutation, and truncating mutation group were 94%, 61%, and 43%, respectively. The *TP53* mutation group displayed significantly worse DSS and overall survival rates than the wild-type group (*P* = 0.01 and *P* = 0.007, respectively). Multivariate analyses revealed that the presence of *TP53* mutations and ≥4 metastatic lymph nodes were independent adverse prognostic factors for HPSCC. p53 immunopositivity was detected in 22 patients, including 5 (28%) and 17 (71%) patients in the wild-type and missense mutation groups, whereas none of the patients with truncating mutation exhibited p53 immunopositivity (*P* = 0.0001).

**Conclusion:**

The *TP53* mutation status correlated with poor prognosis in surgically treated HPSCC patients. Specifically, truncating mutations which were not detected by p53 immunohistochemistry were predictive of worst survival.

## Background

Among squamous cell carcinomas (SCC) originating in the upper aerodigestive tract, the management of hypopharyngeal squamous cell carcinoma (HPSCC) remains to be one of the most challenging and controversial topics, due to the poor survival rate and potentially devastating effects on speech and swallowing [1]. Alcohol consumption and acetaldehyde, a toxic product of ethanol metabolism, are widely known as carcinogen of head and neck SCC (HNSCC) and esophageal SCC (ESCC). The activity of *aldehyde dehydrogenase 2*, a key enzyme in the elimination of aldehyde, is reduced by the germline polymorphism Glu504Lys (previously described as Glu487Lys), which is prevalent in Mongoloid but not in Caucasoid or Negroid populations [2]. Therefore, this different genetic background is considered as a major reason of high HPSCC and ESCC incidence rates in East Asia [3, 4].

Tumor suppressor gene *TP53* is the most frequently mutated gene in human cancers: more than 50% of human cancers contain somatic mutations in this gene [5, 6]. Tumor suppressor p53, encoded by the *TP53* gene, is a key protein involved in many cellular anticarcinogenic processes such as apoptosis and cell-cycle control [7]; therefore, p53 is widely known as the guardian of the genome [8]. Molecular alterations in carcinogenesis of HNSCC include loss of p53 function, which is mediated by genetic mechanisms such as *TP53* mutations [9] and loss of heterozygosity [10], or degradation of p53 meditated by the human papillomavirus (HPV) oncoprotein E6 [11].

Two studies previously demonstrated the association between *TP53* mutations and prognosis in surgically treated HNSCC patients. [12, 13] However, these studies did not examine these associations based on the anatomical location of the HNSCCs. Moreover, patients with oropharyngeal SCC (OPSCC) comprised the majority of the cases, and there were a total of only two patients with HPSCC in the two studies. HPV-related OPSCCs commonly express wild type *TP53* [14], creating a potential confounder as HPV-related tumors have a generally favorable prognosis. In contrast, HPV-driven HPSCC is considered rare [15] and the prognostic significance of *TP53* mutation status in HPSCC has not yet been investigated. The aim of this study was to evaluate the prognostic significance of *TP53* mutation status among surgically treated HPSCC patients in Japan, where the HPSCC incidence rate is high.

## Methods

We retrospectively reviewed the clinical charts of HPSCC patients, who had been surgically treated between 2008 and 2014 at the University of Tokyo Hospital. We excluded patients, who underwent salvage surgery after the definitive radiotherapy (RT) or chemoradiotherapy (CRT), and those who received preoperative chemotherapy. We identified 57 HPSCC patients (55 men and 2 women; age range: 46–84 years, median age: 68 years) who underwent initial surgery of primary lesions. Subsites of primary tumor were the pyriform sinus, posterior wall, and postcricoid region, in 37 (65%), 15 (26%), and 5 (9%) patients, respectively. TNM staging was done according to the 7th edition of the Union for International Cancer Control (2009) staging guidelines. The indication for postoperative RT/CRT was comprehensively determined on the basis of the clinicopathological status of the patients including impaired performance status, inadequate surgical margin, ≥4 metastatic LNs, presence of extranodal extension (ENE), and postoperative complications as well as the consent of patient. The Institutional Review Board of the University of Tokyo Hospital approved this study (#2487 and #2904).

### Determination of human papillomavirus status

In OPSCC, p16 immunopositivity is commonly used as a surrogate marker for HPV determination [16]. Therefore, the p16 status was evaluated in surgically excised specimens using immunohistochemistry (IHC) according to the standard techniques as previously described [17]. A mouse p16 monoclonal antibody (1:100 dilution; Santa Cruz Biotechnology, CA, USA) was used as the primary antibody, and immunostained samples were blindly reviewed and scored independently by two investigators (M. A and Y. S). In accordance with previous studies, p16 positivity by IHC was defined as strong and diffuse nuclear and cytoplasmic staining in ≥70% of the tumor cells [16, 17].

However, p16 expression does not always indicate the presence of HPV DNA, and the combination of p16 expression determined by IHC with HPV DNA determination by polymerase chain reaction (PCR) or in situ hybridization (ISH) is considered to provide the almost perfect sensitivity and specificity [18, 19]. Therefore, p16-immunopositive specimens were also tested for HPV DNA by HPV-ISH, as previously described [19, 20]. Briefly, HPV DNA was detected using an ISH method with catalyzed signal amplification (GenPoint signal amplification system; Dako, Kyoto, Japan), in accordance with the manufacturer’s instructions. Slides were hybridized using a biotinylated GenPoint HPV probe (This probe has been found to react with HPV types 16, 18, 31, 33, 35, 39, 45, 51, 52, 56, 58, 59, and 68 on FFPE tissues and/or cells by ISH, Dako). Slides were scored as positive for HPV if a punctate signal pattern was observed in almost all tumor nuclei.

### Genomic DNA extraction

Tumor tissue specimens were collected during surgery, and snap-frozen in liquid nitrogen and stored at −80 °C. Genomic DNA was extracted using the QIAamp DNA Mini Kit (Qiagen, Hilden, Germany), in accordance with the manufacturer’s protocol. In specimens where the harvest of frozen sections appeared to interfere with the pathological margins, DNA was isolated from formalin-fixed, paraffin-embedded (FFPE) tissue blocks. Briefly, the tumor lesions on hematoxylin and eosin-stained slides were marked, and the corresponding areas were identified on unstained tissue sections. Each selected area was carefully dissected under microscopic observation. Genomic DNA was then extracted using the QIAamp DNA FFPE Tissue Kit (Qiagen).

### Detection of *TP53* mutations

PCR amplification and Sanger sequencing were performed to detect *TP53* mutations in exons 2–9, containing 98% of all mutations described in HNSCC cases [21]. A total of 20 ng/μl genomic DNA per sample was used for PCR amplification using PrimeSTAR HS DNA Polymerase(Takara Bio, Shiga, Japan). Amplification conditions included two-step cycle of 98 °C for 15 s and 68 °C for 90 s, for a total of 44 cycles, except for the amplification of exon 2–3 fragments harvested from frozen and FFPE specimens and exon 6 fragments harvested from FFPE specimens, which were amplified by nested PCR (25 cycles each) using two primer pairs. Subsequently, PCR products harvested from FFPE tissue were purified using the QIAquick PCR Purification Kit (Qiagen), in accordance with the manufacturer’s protocol. Mutations were confirmed by Sanger sequencing using the Big Dye Terminator v3.1 Cycle Sequencing Kit and 3130xL Genetic Analyzer (Applied Biosystems, CA, USA). In this study, nonsense mutations, splice variants, and frameshifts were defined as truncating mutations, that lead to nonfunctional p53, based on previous studies [13, 22]. All samples were sequenced twice with independent PCR using forward and reverse primers.

### Immunohistochemistry for p53 expression

IHC for p53 expression was performed according to standard IHC techniques. A mouse p53 monoclonal antibody clone DO-7 (1:100 dilution; Leica Biosystems, Nussloch, Germany) was used as the primary antibody. In accordance with a previous study, a sample was determined as p53-immunopositive when ≥10% of tumor nuclei were immunostained [23].

### Statistical analyses

Primary endpoint was disease-specific survival (DSS) and secondary endpoint was overall survival (OS). Potential correlations between the treatment method and several clinical features were evaluated using the chi-square test; for analyses in which there were <4 patients, the Fisher’s exact test was used. Survival was analyzed using the Kaplan–Meier method and the log-rank test. Variables were also analyzed by multivariate survival analysis using the Cox proportional hazards model. Hazard ratios (HR) and 95% confidence intervals (CI) were calculated to determine the effect of each variable on outcomes. *P* values <0.05 were considered statistically significant. GraphPad Prism software version 5 (GraphPad Software, CA, USA) was used for the chi-square, Fisher’s exact, Mann-Whitney’s *U*, and log-rank tests. Mac Tahenryo-Kaiseki version 2.0 (ESUMI, Tokyo, Japan) was used for multivariate Cox regression models.

## Results

### Human papillomavirus status of patients with hypopharyngeal squamous cell carcinoma

**Fig. 1:**
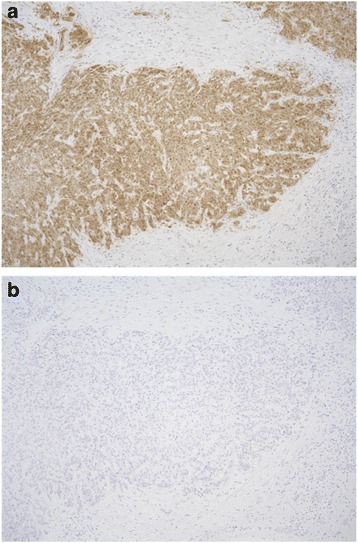
The representative case of p16-immunopositive tumor. **a** p16 immunostaining, and **b** HPV-ISH analysis of the identical tumor. None of p16-immunopositive tumor in our HPSCC cohort was positive by HPV-ISH (original magnification × 100)

In this cohort of 57 HPSCC patients, 3 (5%) patients were immunopositive for p16; however, none of these three patients had detectable HPV DNA by HPV-ISH. Therefore, HPSCC was confirmed to be unrelated to HPV in all patients in this study (Fig. 1).

### Distribution of *TP53* mutations

**Fig. 2:**
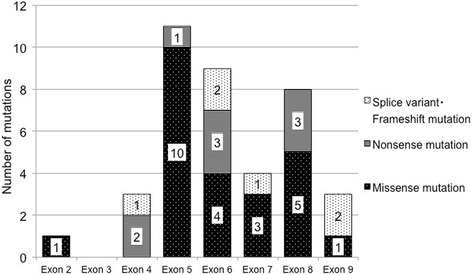
Distribution of *TP53* mutations according to the affected exons. Exon 2, 3, 4, 5, 6, 7, 8, and 9 of *TP53* mutations were found in 1, 0, 3, 11, 9, 4, 8, and 3 patients, respectively

*TP53* mutations were detected in 39 (68%) patients. Missense mutations, nonsense mutations, splicing variants, and frameshift mutations were found in 24 (42%), 9 (16%), 4 (7%), and 2 (3%) patients, respectively. *TP53* mutations in exon 2, 3, 4, 5, 6, 7, 8, and 9 were found in 1, 0, 3, 11, 9, 4, 8, and 3 patients, respectively (Fig. 2).

### Clinicopathological features and *TP53* mutation status

Table 1 summarizes the clinical data and *TP53* status. Table 2 shows the clinicopathological features according to *TP53* mutation status. Histopathological analysis revealed positive surgical margins in 11 (19%) patients, ≥4 metastatic LNs in 14 (25%) patients, and ENE in 15 (26%) patients. Postoperative RT/CRT was administered to 16 (28%) patients. Of note, all of stage I/II patients (5 patients) had wild-type *TP53*, and patients with a past history of HNSCC or ESCC were significantly greater in the *TP53* mutation groups than in the wild-type groups (*P* = 0.02). Administration of postoperative RT/CRT did not correlate with *TP53* mutation status (*P* = 0.25).

**Table 1:** Clinical data and *TP53* status

Age, gender	TN	*TP53*	p53-IHC	No. of LNs	Tumor differentiation	Margins	ENE	Follow-up periods (months)	
62 M	T3N2b	truncating	–	2	M	+	–	101	NED
73 M	T3N2b	wt	+	9	M	+	–	85	DOC
78 M	T3 N0	truncating	–	0	M	–	–	11	DOD
49 M	T2 N0	wt	+	0	W	–	–	94	NED
72 M	T3 N1	missense	+	2	W	–	–	36	DOC
65 M	T3N2b	missense	+	3	M	–	+	27	NED
68 M	T2N2b	wt	–	11	W	–	–	92	NED
67 M	T3N2b	truncating	–	5	M	+	–	11	DOD
81 M	T3 N1	wt	–	3	W	–	–	66	NED
80 M	T3N2b	truncating	–	3	M	–	+	17	DOD
64 M	T1N2b	truncating	–	2	W	–	+	82	NED
79 M	T3 N0	missense	–	0	M	–	–	66	NED
81 M	T3N2b	truncating	–	2	M	–	+	24	DOD
66 M	T4aN0	truncating	–	0	M	–	–	76	NED
81 M	T3 N0	missense	+	0	W	–	–	4	DOC
62 M	T2 N0	wt	–	1	P	–	–	75	NED
83 M	T1 N0	wt	–	0	P	+	–	41	DOD
70 M	T3N2b	truncating	–	14	M	–	+	11	DOD
75 M	T2N2b	wt	+	2	M	+	–	55	NED
55 M	T4aN2b	missense	+	4	W	–	+	24	DOD
46 M	T4aN0	missense	+	0	M	–	–	66	DOC
55 M	T4aN2b	missense	–	6	W	–	–	12	NED
71 M	T3N2c	missense	+	25	M	–	+	17	DOD
79 M	T4aN0	missense	+	1	M	–	–	7	DOD
63 M	T3 N0	missense	–	2	M	–	–	29	DOD
67 M	T2 N0	wt	–	0	W	–	–	60	NED
61 M	T4aN2b	missense	+	4	W	+	+	5	DOD
84 M	T4aN0	truncating	–	0	M	–	–	8	DOD
60 M	T4aN2c	truncating	–	12	P	–	–	9.2	DOC
68 M	T3 N0	truncating	–	0	M	–	–	55	NED
60 M	T1 N0	wt	–	0	M	–	–	53	NED
67 M	T4aN0	wt	–	4	W	–	–	50	NED
72F	T3 N0	missense	+	0	M	–	–	27	NED
72 M	T3 N0	missense	–	0	M	–	–	49	NED
81 M	T3 N1	wt	–	3	W	–	–	17	DOD
72 M	T3 N1	missense	+	1	M	–	–	49	NED
84 M	T3 N0	missense	+	2	M	+	–	5	DOD
47 M	T3 N0	wt	–	0	W	–	–	23	DOC
69 M	T1N2a	missense	–	2	W	+	+	6	DOD
64F	T3N2c	missense	+	3	M	–	–	14	DOD
69 M	T3 N1	missense	–	2	W	–	+	45	NED
64 M	T3 N0	missense	+	0	M	+	–	43	NED
63 M	T3 N1	truncating	–	3	M	–	–	41	NED
76 M	T3 N0	wt	+	1	M	–	+	37	NED
71 M	T4aN2b	missense	+	1	M	–	+	16	DOC
75 M	T3N2b	missense	–	3	M	–	+	37	NED
67 M	T4aN2b	wt	–	4	M	–	–	36	NED
74 M	T3 N0	wt	–	3	P	–	–	42	NED
82 M	T4aN2b	missense	+	1	M	–	–	32	NED
52 M	T3N2c	wt	+	3	M	–	+	32	NED
64 M	T4aN0	missense	+	0	P	–	–	24	DOC
48 M	T4aN2b	truncating	–	4	W	–	–	30	NED
71 M	T4aN0	wt	–	3	M	+	–	28	NED
64 M	T4aN2b	truncating	–	2	M	–	+	17	DOD
84 M	T3N2b	truncating	–	14	M	–	–	16	DOD
64 M	T3 N1	missense	+	1	M	+	–	25	NED
79 M	T4aN1	wt	–	1	W	–	–	24	NED

*IHC* immunohistochemistory, *No. LNs* the number of metastatic lymph nodes, *ENE* extranodal extension, *M* male, *F* female, *W* well differentiated, *M* moderately differentiated, *P* poorly differentiated, *NED* no evidence of disease, *DOD* died of the disease, *DOC* died of other cause

**Table 2:** Clinicopathological parameters according to *TP53* mutation status

Clinicopathological features		Total (*n* = 57)	*TP53* status		*P* value
			Wild-type (*n* = 18)	Mutation (*n* = 39)	
Age	Range (median)		47–83 (70)	46–84 (68)	0.45**
Subsites	PS	27	12	15	0.25
	PC	5	0	5	
	PW	15	6	9	
T	T1–3	40	14	26	0.30*
	T4	17	4	13	
N	N0/1	32	13	19	0.15
	N2/3	25	5	20	
Stage	I/II	5	5	0	0.002*
	III/IV	52	13	39	
Tumor differentiation	W./M. SCC	52	15	37	0.31*
	P. SCC	5	3	2	
Margin	Negative	46	14	32	0.73
	Positive	11	4	7	
No. of metastatic LNs	≤3	43	14	29	1.00
	≥4	14	4	10	
ENE	Absent	42	16	26	0.11*
	Present	15	2	13	
PORT/CRT	Absent	41	14	27	0.25
	Present	16	4	12	
Anamnestic SCC	Absent	42	17	25	0.02*
	Present	15	1	14	

*PS* pyriform sinus, *PC* postcricoid, *PW* posterior wall, *T* tumor classification, *N* nodal classification, *Stage* stage classification, *W* well differentiated, *M* moderately differentiated, *P* poorly differentiated, *SCC* squamous cell carcinoma, *No* number, *LN* lymph node, *ENE* extranodal extension, *PORT/CRT* postoperative radiotherapy/chemoradiotherapy, *Anamnestic SCC* anamnestic squamous cell carcinoma arising from esophagus and head and neck region, *: Fisher’s exact tests were used. **: Mann-Whitney’s U test was used

### Association between *TP53* mutation and p53 expression

**Fig. 3:**
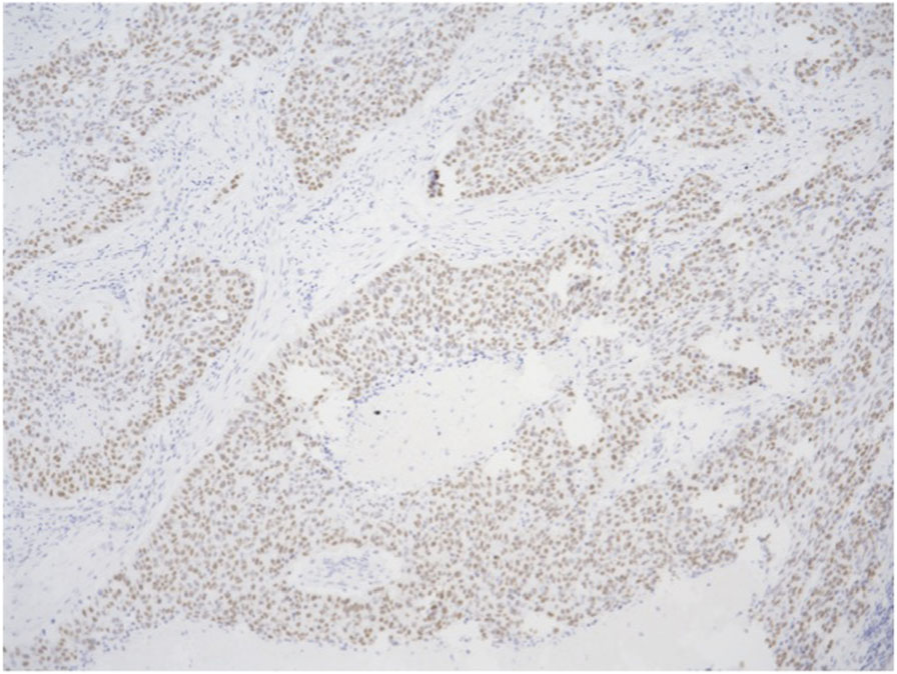
Representative images of p53-immunopositive tumor (original magnification × 100)

Representative images of specimens exhibiting p53 immunopositivity are presented in Fig. 3. p53 immunopositivity was detected in 22 patients, including 5 (28%) and 17 (71%) patients in the wild-type and missense mutation groups, whereas there were no patients with p53 immunopositivity in the truncating mutation group (*P* = 0.0001, chi-square test).

### Correlation between *TP53* mutation status and prognosis

**Fig. 4:**
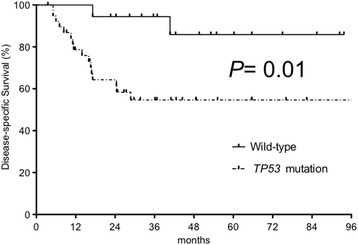
Disease-Specific Survival (DSS) according to the presence of *TP53* mutation. The 3-year DSS rate of patients with wild-type *TP53* was significantly longer than that of patients with *TP53* mutations (94% vs 55%, *P* = 0.01)

**Fig. 5:**
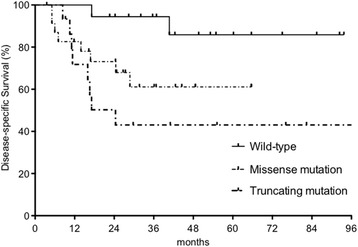
Disease-Specific Survival (DSS) according to the *TP53* mutation status. The 3-year DSS rate of the patients with wild-type *TP53*, missense *TP53* mutation, and truncating *TP53* mutation were 94%, 61%, and 43%, respectively

Eighteen (32%) patients died from HPSCC, whereas 8 (14%) patients died from other causes. The remaining 31 (54%) patients were alive and disease-free on last follow-up date. The median follow-up period for the entire cohort was 29 months (range: 3.5–101 months), whereas 45 months (range: 24–101 months) for patients who survived (*n* = 31) and 16 months (range: 3.5–85 months) for those who died (*n* = 26). The 3-year DSS of the wild-type group was significantly longer than that of the *TP53* mutation group (94% vs 55%; *P* = 0.01, Fig. 4). Furthermore, patients with wild-type/missense mutations had significantly better 3-year DSS than those with truncating mutations (76% vs 43%; *P* = 0.03). The 3-year DSS rate of wild-type, missense mutation, and truncating mutation groups were 94%, 61%, and 43%, respectively (Fig. 5). The 3-year OS of the wild-type group was significantly longer than that of the *TP53* mutation group (89% vs 42%; *P* = 0.007). The 3-year OS rate of wild-type/missense mutation group was not significantly different than that of the truncating mutation group (66% vs 40%; *P* = 0.14). The 3-year OS rate of the wild-type, missense mutation, and truncating mutation group were 89%, 43%, and 40%, respectively. In contrast, p53 immunopositivity did not correlate with DSS (*P* = 0.77). In the subgroup analyses of 52 stage III/IV patients, the 3-year DSS of the wild-type group was significantly longer than that of the *TP53* mutation group (92% vs 55%; *P* = 0.02). The 3-year OS of the wild-type group was significantly longer than that of the *TP53* mutation group (92% vs 42%; *P* = 0.006).

Table 3 shows the associations between the clinicopathological factors and DSS in univariate analysis. The presence of ≥4 metastatic LNs (*P* = 0.04) and ENE (*P* = 0.03) were poor prognostic factors. In contrast, tumor differentiation grade, T classification, stage, surgical margin, and postoperative RT/CRT did not correlate with DSS.

**Table 3:** Univariate analyses for disease-specific survival

Variables		HR (95% CI)	*P* value
Tumor differentiation	W./M. vs. P.SCC	0.63 [0.13–3.09]	0.57
T classification	T1–3 vs. T4	1.12 [0.39–3.25]	0.83
Stage	Stage I/II vs. III/IV	2.30 [0.55–9.56]	0.25
Margin	Negative vs. Positive	2.08 [0.61–7.07]	0.24
No. of metastatic LNs	≤3 vs. ≥4	3.34 [1.04–10.7]	0.04
ENE	Absent vs. Present	3.34 [1.11–10.1]	0.03
PORT/CRT	Performed vs. Not performed	0.91 [0.33–2.53]	0.86
*TP53* mutation	Wild-type vs. Mutation	3.32 [1.28–8.60]	0.01
p53 immunopositivity	Negative vs. Positive	0.87 [0.33–2.27]	0.77

*HR* hazard ratio, *95% CI* 95% confidence interval, *W* well differentiated, *M* moderately differentiated, *P* poorly differentiated, *SCC* squamous cell carcinoma, *T* tumor classification, *No* number, *LN* lymph node, *ENE* extranodal extension, *PORT/CRT* postoperative radiotherapy/chemoradiotherapy

Multivariate Cox proportional hazard analysis using variables based on univariate analyses was conducted to determine independent prognostic factors for DSS and OS. The presence of *TP53* mutations (*P* = 0.04; HR, 4.96; 95% CI, 1.08–22.8, and *P* = 0.02; HR, 4.75; 95% CI, 1.35–16.7, respectively) and ≥4 metastatic LNs (*P* = 0.03; HR, 3.00; 95% CI, 1.12–8.04, and *P* = 0.02; HR, 2.89; 95% CI, 1.22–6.86, respectively) have significant adverse effects on both DSS and OS. In the subgroup analyses of 52 stage III/IV patients, the presence of *TP53* mutations was a significant adverse prognostic factor on OS, and nearly reached significance on DSS. (Table 4).

**Table 4:** Multivariate analyses for disease-specific and overall survival

Variables		Overall (*n* = 57)				Stage III/IV (*n* = 52)			
		DSS		OS		DSS		OS	
		HR [95% CI]	*P* value	HR [95% CI]	*P* value	HR [95% CI]	*P* value	HR [95% CI]	*P* value
*TP53*	Wild-type vs. Mutation	4.96 [1.08–22.8]	0.04	4.75 [1.35–16.7]	0.02	7.72 [0.98–60.7]	0.05	5.51 [1.24–24.5]	0.03
No. of metastatic LNs	≤3 vs. ≥4	3.00 [1.12–8.04]	0.03	2.89 [1.22–6.86]	0.02	3.07 [1.13–8.35]	0.03	2.89 [1.20–6.94]	0.02
ENE	Absence vs. Presence	1.85 [0.70–4.86]	0.21	1.39 [0.59–3.27]	0.45	1.81 [0.68–4.80]	0.23	1.36 [0.58–3.21]	0.48

*DSS* disease-specific survival, *OS* overall survival, *HR* hazard ratio, *95% CI* 95% confidence interval, *No* the number, *ENE* extranodal extensio

## Discussion

In this retrospective study, we demonstrated that the *TP53* mutation status significantly correlated with poor prognosis in surgically treated HPSCC patients. Specifically, patients with truncating mutations exhibited the worst prognosis. To the best of our knowledge, this is the first study focusing on the association between *TP53* mutation status and prognosis of HPV-unrelated HPSCC. The result of the current study was consistent with the previous studies investigating all HNSCC subsites [12, 13], which included patients with HPV-driven OPSCC.

HPSCC is rarely caused by HPV-driven carcinogenesis as we confirmed in the current study and occurs more frequently in East Asian population than in other regions of the world. Survival of patients with HPSCC has not markedly improved in recent decades. In the last two-decades, CRT and induction chemotherapy followed by RT have become the option for advanced HNSCC patients who prefer nonsurgical organ preservation [24–26]. However, RT-induced late toxicity, such as dysphasia and osteonecrosis distresses emerging issues for cancer survivors. The recent development of minimally invasive surgical procedures, such as transoral robotic surgery (TORS) and transoral videolaryngoscopic surgery (TOVS) techniques, has broadened surgical indications and appeared to result in better outcomes with respect to the postoperative speech and swallowing function [27, 28]. Therefore, surgery remains the main treatment modality for HPSCC patients. Our multivariate analyses revealed that both the *TP53* mutation status and the presence of ≥4 metastatic LNs were independent adverse prognostic factors for surgically treated HPSCC patients. In the previous study, we demonstrated that the presence of multiple metastatic LNs was significantly associated with the poor prognosis and the incidence of distant metastases in advanced HPSCC patients treated with total pharyngolaryngectomy [29]. Collectively, *TP53* mutations can be a useful biomarker for HPSCC patients, in addition to the traditional metastatic LN number.

Interestingly, the past history of HNSCC or ESCC was significantly higher in the *TP53* mutation group than in the wild-type group in the present study. HNSCC and ESCC have been known to occur synchronously or metachronously, which might be explained by the concept of “field cancerization” first introduced by Slaughter et al. in 1953 [30]. Currently, repetitive exposure to acetaldehyde is considered to play a key role in field cancerization of the squamous epithelium in the head and neck region and the esophagus [31]. Moreover, Waridel et al. reported that mutations in *TP53* were frequent and early events in the pathogenesis of HNSCC and identified the expansion of multiple clones of mutant p53-containing cells as an important biological step in field cancerization [32]. Our findings in the current study led further support to these observations. Future studies with larger sample size and longitudinal evaluations, supported with basic research, are necessary to confirm this hypothesis.

In the current study, we demonstrated that p53 immunopositivity was observed most frequently in the presence of missense mutations. Wild-type p53 protein is rapidly degraded via the ubiquitin-proteasome system, resulting in low p53 protein expression. Conversely, the nonsense-mediated RNA decay and the resultant decreased amount of the protein considered to be the reason why truncating p53 proteins cannot be detected by IHC [33]. Some missense mutations, that result in increased p53 immunopositivity can lead to a dominant-negative or a gain-of-function phenotype [34, 35], Our observations in the current study support these biological mechanisms; therefore, the distinction between missense and truncating mutations is reasonable for the clinical categorization of the *TP53* mutation status.

The Cancer Genome Atlas (TCGA) reported that *TP53* mutations were detected in 84% of HPV-unrelated HNSCC cases using whole-exome sequencing analysis [14]. In comparison, the frequency of *TP53* mutations was lower in the HPSCC cohort of the current study, which might be partially due to differences in racial composition and tumor subsites. The HNSCC cohort of TCGA consisted almost entirely of Caucasoid and Negroid populations, with only two HPSCC patients. Additionally, it is possible that mutation detection sensitivity of whole-exome sequencing was superior to that of Sanger sequencing.

To improve the prognoses of HPSCC patients with *TP53* mutations, adjuvant therapy should be selectively administered to these patients. *TP53* mutation, however, is also known as a predictive marker for chemo- and radioresistance in HNSCCs. [36, 37] Therefore, it might be unreasonable to use *TP53* mutation status as a therapeutic biomarker for existing postoperative treatments including RT/CRT. Although most of the current targeted therapies are inhibitors of oncogenic pathways, development of p53-targeted therapy is warranted.

One of the limitations of our study was a lack of detailed comparison and functional study of each *TP53* mutations, due to the small sample size. In line with previous reports on HNSCC [13], various mutation types were detected in various regions of *TP53* genes. Further investigation with larger sample size is required to elucidate the potential associations between the mutations with respect to functional and biological effects and prognosis. Furthermore, the number of T1–2 tumors was small in this study. Recently, TORS and TOVS techniques for T1–2 tumors to which RT/CRT was previously preferable were broadened. Therefore, further accumulation of T1–2 patients is also required.

## Conclusions

We demonstrated that *TP53* mutations had a significant impact on prognosis, in surgically treated HPSCC patients. In particular, truncating mutations which were not detected by p53 IHC were shown to have predictive value for a worst survival. Further confirmation from prospective studies with larger sample size including more T1–2 patients is warranted.

## Abbreviations

CRT: Chemoradiotherapy
ENE: Extranodal extension
ESCC: Esophageal squamous cell carcinoma
FFPE: Formalin-fixed, paraffin-embedded
HNSCC: Head and neck squamous cell carcinoma
HPSCC: Hypopharyngeal squamous cell carcinoma
HPV: Human papillomavirus
IHC: Immunohistochemistry
ISH: In situ hybridization
LN: Lymph node
ND: Neck dissection
OPSCC: Oropharyngeal squamous cell carcinoma
PCR: Polymerase chain reaction
RT: Radiotherapy
SCC: Squamous cell carcinoma
TCGA: The Cancer Genome Atlas
TORS: Transoral robotic surgery
TOVS: Transoral videolaryngoscopic surgery

## References

[1] Montgomery PQ, Phys Evan PH, Gullane PJ (2009). Principles and practice of head and neck surgery and oncology.

[2] Goedde HW, Agarwal DP, Fritze G, Meier-Tackmann D, Singh S, Beckmann G (1992). Distribution of ADH2 and ALDH2 genotypes in different populations. Hum Genet.

[3] Hamajima N, Takezaki T, Tajima K (2002). Allele frequencies of 25 polymorphisms pertaining to cancer risk for Japanese, Koreans and Chinese. Asian Pac J Cancer Prev.

[4] Asakage T, Yokoyama A, Haneda T, Yamazaki M, Muto M, Yokoyama T (2007). Genetic polymorphisms of alcohol and aldehyde dehydrogenases, and drinking, smoking and diet in Japanese men with oral and pharyngeal squamous cell carcinoma. Carcinogenesis.

[5] Levine AJ (1997). p53, the cellular gatekeeper for growth and division. Cell.

[6] Toledo F, Wahl GM (2006). Regulating the p53 pathway: in vitro hypotheses, in vivo veritas. Nat Rev Cancer.

[7] Vogelstein B, Lane D, Levine AJ (2000). Surfing the p53 network. Nature.

[8] Efeyan A, Serrano M (2007). p53: guardian of the genome and policeman of the oncogenes. Cell Cycle.

[9] Olshan AF, Weissler MC, Pei H, Conway K (1997). p53 mutations in head and neck cancer: new data and evaluation of mutational spectra. Cancer Epidemiol Biomark Prev.

[10] Gonzalez MV, Pello MF, Lopez-Larrea C, Suarez C, Menendez MJ, Coto E (1995). Loss of heterozygosity and mutation analysis of the p16 (9p21) and p53 (17p13) genes in squamous cell carcinoma of the head and neck. Clin Cancer Res.

[11] Scheffner M, Werness BA, Huibregtse JM, Levine AJ, Howley PM (1990). The E6 oncoprotein encoded by human papillomavirus types 16 and 18 promotes the degradation of p53. Cell.

[12] Poeta ML, Manola J, Goldwasser MA, Forastiere A, Benoit N, Califano JA (2007). TP53 mutations and survival in squamous-cell carcinoma of the head and neck. N Engl J Med.

[13] Lindenbergh-van der Plas M, Brakenhoff RH, Kuik DJ, Buijze M, Bloemena E, Snijders PJ (2011). Prognostic significance of truncating *TP53* mutations in head and neck squamous cell carcinoma. Clin Cancer Res.

[14] The Cancer Genome Atlas Network (2015). Comprehensive genomic characterization of head and neck squamous cell carcinomas. Nature.

[15] Ang KK, Harris J, Wheeler R, Weber R, Rosenthal DI, Nguyen-Tân PF (2010). Human papillomavirus and survival of patients with oropharyngeal cancer. N Engl J Med.

[16] Gillison ML, Zhang Q, Jordan R, Xiao W, Westra WH, Trotti A (2012). Tobacco smoking and increased risk of death and progression for patients with p16-positive and p16-negative oropharyngeal cancer. J Clin Oncol.

[17] Saito Y, Yoshida M, Ushiku T, Omura G, Ebihara Y, Shimono T (2013). Prognostic value of p16 expression and alcohol consumption in Japanese patients with oropharyngeal squamous cell carcinoma. Cancer.

[18] Thavaraj S, Stokes A, Guerra E, Bible J, Halligan E, Long A (2011). Evaluation of human papillomavirus testing for squamous cell carcinoma of the tonsil in clinical practice. J Clin Pathol.

[19] Saito Y, Yoshida M, Omura G, Kobayashi K, Fujimoto C, Ando M (2015). Prognostic value of p16 expression irrespective of human papillomavirus status in patients with oropharyngeal carcinoma. Jpn J Clin Oncol.

[20] Saito Y, Ebihara Y, Ushiku T, Omura G, Kobayashi K, Ando M (2014). Negative human papillomavirus status and excessive alcohol consumption are significant risk factors for second primary malignancies in Japanese patients with oropharyngeal carcinoma. Jpn J Clin Oncol.

[21] Bouaoun L, Sonkin D, Ardin M, Hollstein M, Byrnes G, Zavadil J, Olivier M. TP53 Variations in Human Cancers: New Lessons from the IARC TP53 Database and Genomics Data. Hum Mutat. 2016;37(9):865–76. 10.1002/humu.23035 27328919

[22] Oliver M, Langer A, Carrieri P, Bergh J, Klaar S, Eyfjord J (2006). The clinical value of somatic *TP53* gene mutations in 1,794 patients with breast cancer. Clin Cancer Res.

[23] Rodrigo JP, Martínez P, Allonca E, Alonso-Durán L, Suárez C, Astudillo A (2014). Immunohistochemical markers of distant metastasis in laryngeal and hypopharyngeal squamous cell carcinomas. Clin Exp Metastasis.

[24] Pignon JP, Bourhis J, Domenge C, Designe L (2000). Chemotherapy added to locoregional treatment for head and neck squamous-cell carcinoma: three meta-analyses of updated individual data. MACH-NC collaborative group. Meta-analysis of chemotherapy on head and neck cancer. Lancet.

[25] Posner MR, Hershock DM, Blajman CR, Mickiewicz E, Winquist E, Gorbounova V (2007). Cisplatin and fluorouracil alone or with docetaxel in head and neck cancer. N Engl J Med.

[26] Posner MR, Norris CM, Wirth LJ, Shin DM, Cullen KJ, Winquist EW (2009). Sequential therapy for the locally advanced larynx and hypopharynx cancer subgroup in TAX 324: survival, surgery, and organ preservation. Ann Oncol.

[27] Park YM, Kim YS, De Virgilio A, Lee SY, Seol JH, Kim SH (2012). Transoral robotic surgery for hypopharyngeal squamous cell carcinoma: 3-year oncologic and functional analysis. Oral Oncol.

[28] Tomifuji M, Araki K, Yamashita T, Shiotani A (2014). Transoral videolaryngscopic surgery for oropharyngeal, hypopharyngeal, and supraglottic cancer. Eur Arch Otorhinolaryngol.

[29] Omura G, Ando M, Saito Y, Kobayashi K, Yamasoba T, Asakage T (2015). Disease control and clinicopathological prongostic factors of total pharyngolaryngectomy for hypopharyngeal cancer: a single-center study. Int J Clin Oncol.

[30] Slaughter DP, Southwick HW, Smejkal W (1953). Field cancerization in oral stratified squamous epithelium; clinical implications of multicentric origin. Cancer.

[31] Ohashi S, Miyamoto S, Kikuchi O, Goto T, Amanuma Y, Muto M (2015). Recent advances from basic and clinical studies of esophageal squamous cell carcinoma. Gastroenterology.

[32] Waridel F, Estreicher A, Bron L, Flaman JM, Fontolliet C, Monnier P (1997). Field cancerisation and polyclonal p53 mutation in the upper aero-digestive tract. Oncogene.

[33] Ebihara Y, Iwai M, Akashi K, Ito T, Omura G, Saito Y (2014). High incidence of null-type mutations of the *TP53* gene in Japanese patients with head and neck squamous cell carcinoma. J Cancer Ther.

[34] Petitjean A, Achatz MIW, Borresen-Dale AL, Hainaut P, Olivier M (2007). *TP53* mutations in human cancers: functional selection and impact on cancer prognosis and outcomes. Oncogene.

[35] Xu Y (2008). Induction of genetic instability by gain-of-function p53 cancer mutants. Oncogene.

[36] Perrone F, Bossi P, Cortelazzi B, Locati L, Quattrone P, Pierotti MA (2010). *TP53* mutations and pathologic complete response to neoadjuvant cisplatin and fluorouracil chemotherapy in resected oral cavity squamous cell carcinoma. J Clin Oncol.

[37] Skinner HD, Sandulache VC, Ow TJ, Meyn RE, Yordy JS, Beadle BM (2012). *TP53* disruptive mutations lead to head and neck cancer treatment failure through inhibition of radiation-induced senescence. Clin Cancer Res.

